# Repeatability, variability and reference values of pulsed wave Doppler echocardiographic measurements in healthy Saanen goats

**DOI:** 10.1186/1746-6148-8-190

**Published:** 2012-10-16

**Authors:** Aurélia A Leroux, Frédéric Farnir, Marie L Moonen, Charlotte F Sandersen, Stefan Deleuze, Hélène Amory

**Affiliations:** 1 Equine Clinic, Department of Companion Animals and Equids, Faculty of Veterinary Medicine, University of Liege, Boulevard de Colonster 20, Bât B41, Liege, Sart Tilman, Belgium; 2 Biostatistics, Bioinformatics and Animal Selection, Department of Animal Production, Faculty of Veterinary Medicine, University of Liege, Boulevard de Colonster 20, Bât B43, Liege, Sart Tilman, Belgium; 3 Department of Cardiology, University Hospital of Liege, Medicine Faculty, University of Liege, Avenue de l’Hôpital 13, Bât B35, Liege, Sart Tilman, Belgium

**Keywords:** Goat, Pulsed wave, Doppler echocardiography, Reference intervals, Repeatability, Variability

## Abstract

**Background:**

Pulsed wave (PW) Doppler echocardiography has become a routine non invasive cardiac diagnostic tool in most species. However, evaluation of intracardiac blood flow requires reference values, which are poorly documented in goats. The aim of this study was to test the repeatability, the variability, and to establish the reference values of PW measurements in healthy adult Saanen goats. Using a standardised PW Doppler echocardiographic protocol, 10 healthy adult unsedated female Saanen goats were investigated three times at one day intervals by the same observer. Mitral, tricuspid, aortic and pulmonary flows were measured from a right parasternal view, and mitral and aortic flows were also measured from a left parasternal view. The difference between left and right side measurements and the intra-observer inter-day repeatability were tested and then the reference values of PW Doppler echocardiographic parameters in healthy adult female Saanen goats were established.

**Results:**

As documented in other species, all caprine PW Doppler parameters demonstrated a poor inter-day repeatability and a moderate variability. Tricuspid and pulmonary flows were best evaluated on the right side whereas mitral and aortic flows were best obtained on the left side, and reference values are reported for healthy adult Saanen goats.

**Conclusions:**

PW Doppler echocardiography allows the measurement of intracardiac blood flow indices in goats. The reference values establishment will help interpreting these indices of cardiac function in clinical cardiac cases and developing animal models for human cardiology research.

## Background

During the last thirty years, pulsed wave (PW) Doppler echocardiography has been developed first in humans [1,2], and then in several domestic animals species including dogs [3,4], cats [5], horses [6,7], cattle [8] and sheep [9]. This technique has become a routine for the diagnosis and evaluation of heart disease in veterinary medicine [10]. It allows detecting the returning signal during a time interval specified by a sample depth ignoring all other signals. Blood cells flow moving around the chosen specific location is analysed and gives information about direction, velocity, character and timing of the blood flow which cannot be assess without Doppler imaging [2,10]. So it provides a non-invasive tool to evaluate intracardiac blood flow, to diagnose regurgitant flow through the cardiac valves and intracardiac shunts and to assess systolic and diastolic function of the heart [10,11]. Accurate interpretation of Doppler echocardiographic variables requires reference values following standardised measurement guidelines in the studied species to interpret indices of cardiac function [10].

Goats are animals easy to handle with a body and heart size comparable to that of humans. This makes the goat an attractive candidate for the development of animal models for human cardiology research, especially chronic models relying on measurements in awake or exercising animals [12,13]. Time-motion mode (M-mode), two-dimensional (2D) and colour-flow Doppler echocardiography has been demonstrated to be feasible in Philippine native goats [14] and in a pygmy goat [15]. More recently, M-mode and 2D-echocardiographic reference values have been established in awake healthy Saanen goats after repeatability and variability studies [16] and the effect of general anaesthesia in Saanen goats was studied using 2D- and colour Doppler echocardiography [17]. By contrast, no reference values of PW Doppler parameters were reported in goats. Only one study devoted to evaluate the cardiovascular changes induced by pregnancy and lactation reported PW Doppler values in Swedish adult domestic goats [18]. However, only aortic flow measurements were obtained and repeatability of these measurements was not determined in this study.

The aim of the present study was to test the variability, to establish the best technique to evaluate intracardiac blood flow and to establish the reference values of blood flow PW Doppler measurements in unsedated standing adult Saanen goats.

## Methods

This experimental protocol was a part of a larger study and followed the guidelines of ethics of University of Liege. The reference number from the Ethical Committee was 655 accepted on the 1^st^ of June 2007.

### Animals

Ten adult nulliparous female Saanen goats, aged 22 to 28 months (mean age: 24.7 ± 2.1 months) and weighing 51 to 80 kg (mean body weight: 65.1 ± 8.3 kg) were studied. All animals were considered to be healthy based on the history and the absence of abnormalities on physical examination, cardiac auscultation, electrocardiography (ECG), haematology, measurement of the serum haptoglobin, fibrinogen, total protein content, and the electrophoresis of serum proteins. A complete colour and PW Doppler echocardiography (from right and left side) was also performed in each goat before starting the protocol to be sure that the studied goats were free of any cardiac disease, including any valvulopathy. All the animals were fully accustomed to be handled. Before imaging, the hair was shaved on both sides, from the 3^rd^ to the 5^th^ right intercostal space just caudal to the triceps muscle mass, from 3 to 5 cm below the right olecranon to 5 to 10 cm above it. The shaved areas were then copiously rinsed with water and acoustic coupling was obtained using ultrasound gel.

### Echocardiographic protocol

In the 10 studied goats, the PW Doppler echocardiographic protocol was repeated 3 times at one-day intervals. An ultrasound system (Vivid i, Software version 9.1.0, General Electric Healthcare Europe GMBH, Diegem, Belgium) equipped with colour flow mapping and spectral Doppler mode and with a 1.5-3.6 MHz phased array transducer (GE 3S-RS probe, General Electric Healthcare Europe GMBH, Diegem, Belgium) was used to perform the PW Doppler echocardiography. All examinations and measurements were performed by the same observer (AAL), who had 3 full years expertise in large animal echocardiography (equids, cattle and goats), and were recorded digitally. Recordings were identified using number codes chosen by an external observer. All measurements were blindly performed off line by the same observer (AAL) using specific software (Echo Pac System for Vivid i, Software version 108.1.5, General Electric Healthcare Europe GMBH, Diegem, Belgium). All variables were measured 5 times on 5 different non consecutive cycles and an average of these 5 measurements was calculated for each variable. The cardiac cycle was chosen when the image quality of the flow was optimal. Examinations were performed on standing animals with the right or the left forelimb extended by an assistant as far forward as tolerated by the goat. Heart rate (HR) was calculated from the ECG tracings from 5 successive cardiac cycles during the echocardiographic recordings. Echocardiographic measurements were only performed when HR was less than 120 beats per minute to obviate a stress effect. A colour-flow Doppler echocardiography was performed each day on each measured intracardiac flow to confirm absence of any abnormal shunt.

**Figure 1:**
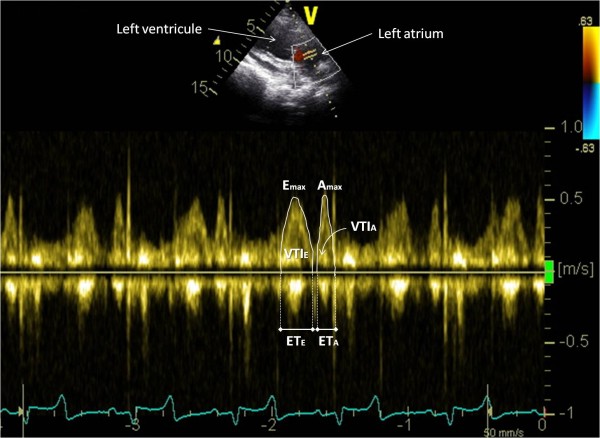
**Mitral inflow velocity spectrum obtained from a left parasternal long axis four chamber view.** The peak early filling velocity (E_max_), the peak filling velocity of the transmitral flow during atrial contraction (A_max_), the E-peak and A-peak velocity time integral (VTI_E_ and VTI_A_ respectively) and the E-peak and A-peak ejection times (ET_E_ and ET_A_ respectively) were measured as shown.

**Figure 2:**
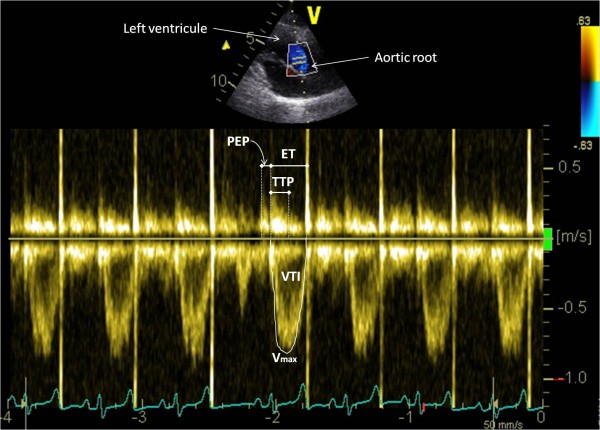
**Aortic flow velocity spectrum obtained from a left parasternal long axis five chamber view.** The peak velocity of the aortic flow (V_max_), the velocity time integral (VTI), the ejection time (ET), the time to peak velocity (TTP) and the pre-ejection period (PEP) of the aortic flow were measured as shown.

Terminology and 2D-image orientation recommended by the Echocardiography Committee of The Specialty of Cardiology, American College of Veterinary Internal Medicine [19], were used. Goats were first examined from the left hemi thorax to assess the aortic and mitral blood flow. The transducer was placed in the 4^th^ intercostal space and adjusted to obtain a long axis four chamber view in 2D mode. From this view, the transducer was angled to optimize spatial alignment between the transducer and the mitral blood flow and the PW mode was selected. The sample volume (4.91mm) was placed between the mitral valve leaflets opening in diastole and the mitral inflow velocity spectrum was recorded (Figure 1). From the initial 2D left parasternal long axis four chambers view, the transducer was then slightly turned forwards to obtain a standard long axis five chamber view. The aortic flow velocity spectrum was obtained by placing the sample volume of the PW mode just downstream from the aortic valve in the aortic root (Figure 2).

**Figure 3:**
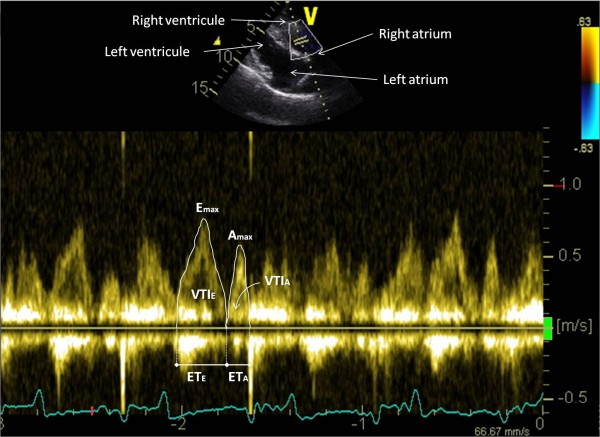
**Tricuspid inflow velocity spectrum obtained from a tilted right parasternal long axis four chamber view.** The peak early filling velocity (E_max_), the peak filling velocity of the tricuspid flow during atrial contraction (A_max_), the E-peak and A-peak velocity time integral (VTI_E_ and VTI_A_ respectively) and the E-peak and A-peak ejection times (ET_E_ and ET_A_ respectively) were measured as shown.

**Figure 4:**
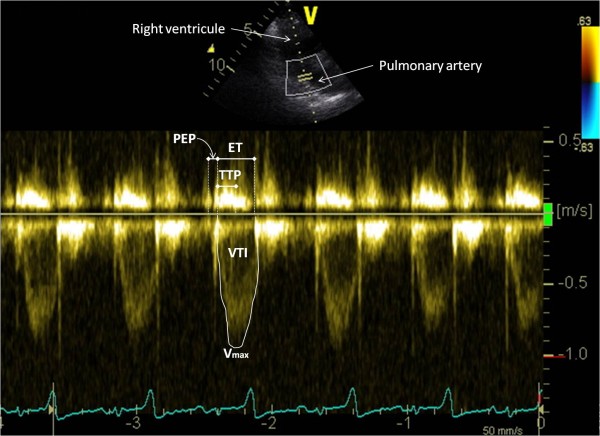
**Pulmonary outflow velocity spectrum obtained from a right parasternal right ventricular inflow-outflow view.** The peak velocity of the pulmonary flow (V_max_), the velocity time integral (VTI), the ejection time (ET), the time to peak velocity (TTP) and the pre-ejection period (PEP) of the pulmonary flow were measured as shown.

The goats were then examined from the right hemi thorax. A right parasternal long axis four chambers view with chordae tendinae and mitral valve clearly visible was obtained in 2D-mode. This view was then tilted as ventrally as possible to obtain the most optimal possible alignment between the transducer and the mitral flow. In PW-mode, the sample volume was placed in the left ventricle just between the mitral valve leaflets opening in diastole to record the mitral inflow velocity spectrum. Then, the transducer was angled to optimize spatial alignment between the transducer and the tricuspid flow and the sample volume was placed in the right ventricle just between the tricuspid valve leaflets opening in diastole to record the tricuspid inflow velocity spectrum (Figure 3). Starting from the right parasternal 2D four chambers view, the transducer was then rotated slightly cranially to obtain a right parasternal long axis view of the left ventricular outflow tract and to measure the aortic internal diameter at the basis of the valves (Ao) at end diastole. From this five chambers view, the beam was tilted in order to obtain an optimal alignment with aortic blood flow. The PW mode was selected and aortic flow velocity spectrum was obtained by placing the sample volume in the aortic root just distal to the valves. The pulmonary artery diameter at end diastole was measured (Pu) from a right parasternal right ventricular inflow-outflow view obtained by directing the transducer slightly cranio-dorsally and by placing it one intercostal space forwards. PW-mode was selected and the sample volume was positioned just distal to the pulmonary valves to assess the pulmonary outflow velocity spectrum (Figure 4).

### Doppler measurements and calculations

From the mitral and tricuspid velocity spectral recordings, the peak velocity during the early ventricular filling called E peak and during the atrial contraction called A peak were measured (E_max_ and A_max_ respectively). The areas under the two velocity peaks (VTI of E peak and of A peak) were measured by manually tracing the modal velocity envelope of the Doppler signal. This allowed the assessment of the mean velocity of the blood flow during the early ventricular filling (E_mean_) and during the atrial contraction (A_mean_). The ejection time (ET) of the E peak and of the A peak were measured from the onset to the end of each peak. The deceleration (Dec) time and slope of the early filling velocity were measured by tracing the slope between the maximal E peak velocity and the return to baseline at the end of the flow profile. From E_max_ and A_max_, the ratio E_max_/A_max_ was calculated.

From the aortic and pulmonary velocity spectral recordings, the peak velocity of the blood flow (V_max_) was measured by placing the cursor at the maximal point of the blood flow profile. The area under the velocity waveform (VTI) was measured by manual tracing of the modal velocity envelope of the flow profile, thus allowing the measurement of the mean velocity of blood flow (V_mean_). The ejection time (ET) was measured from the onset to the end of the spectral waveform, the time to peak (TTP) was measured from the onset of the Doppler waveform to the beginning of the maximum velocity plateau, the pre-ejection period (PEP) was measured from the onset of the QRS complex to the onset of the spectral waveform, and the Dec time was calculated by subtracting TTP from ET. The acceleration (Acc) and Dec slope of the flow profile were measured by tracing the slope from the onset to the peak and from the peak to the end of the velocity spectral waveform, respectively. From the measured parameters, the ratio PEP/ET was calculated and the stroke volume (SV) and the cardiac output (CO) were obtained using the following standard formulae:

(1) $$SV=\pi .VTI.{\left(Ao/2\right)}^{2}$$

(2) CO=SV.HR.

The stoke index (SI) and the cardiac index (CI) were calculated by dividing SV and CO, respectively, by the body weight.

### Repeatability and statistical analysis

In all goats, the echocardiographic protocol was repeated 3 times at one-day intervals by the same observer (AAL). Within each day, each variable was measured 5 times on 5 different non consecutive cardiac cycles. Observed means, standard deviations (SD), least square means, and standard errors (SE) were calculated for each variable. For measurements of tricuspid and pulmonary flows, a two-way ANOVA considering goats, days and interaction between goats and days as factors, and, for measurements of mitral and aortic flows, a three-way ANOVA considering goats, days, side of echocardiography and interaction between these different variables as factors, were performed and allowed the determination of the intra-observer inter-day repeatability of the measurements. A *P*-value less than 0.05 was considered significant. The within-goat within-day variability was evaluated using the coefficient of variation (Within-day CV) measured from standard errors and observed means obtained from the two-way ANOVA for each variable. The within-goat between-day variability (Between-day CV), i.e. the variability of the same repeated measurements on the same goat independently of the day, was evaluated from standard deviations and observed means obtained in a one way ANOVA considering only goats as factor. Degree of variability of each measurement was defined arbitrarly as follows: variables with a CV inferior to 10% were considered to have low variability, those with a CV between 10% and 20% were considered to have moderate variability, and those with a CV superior to 20% were considered to have high variability. In addition to the CV, absolute variability was obtained by calculating confidence interval for each variable. This interval corresponds to the interval within which the real value of the measurement has a 95% chance to be included. Superior and inferior limits of this interval were obtained as follows: observed mean + 1.96xSE and observed mean – 1.96xSE.

For each variable, the mean, the SD, and the range of values for all these measurements were used to establish the reference values of PW Doppler parameters in unsedated adult healthy female Saanen goats.

## Results

The mean HR during the echocardiographic examination was 92.75 ± 11.18 beats/minute and ranged from 75 to 120 beats/minute. The image quality and the Doppler spectra were good in all goats, except for the quality of the tricuspid flow and the right side aortic flow which were often poor. Moreover, obtaining a good quality 2D right parasternal view of the heart base at the level of the pulmonary valves before to shift in PW-mode appeared to be sometimes difficult. For this view, the transducer had to be advanced far forward under the forelimb, which required an assistant pulling the right forelimb forward and upward during the examination.

The least square mean value and the standard error to the mean of each blood flow measurements obtained on day 1, day 2 and day 3 and the multivariable ANOVA test were calculated to evaluate the repeatability of these measurements. Concerning the aortic flow, significant between days differences were observed for V_max_, VTI, AccTime, CO and CI. Almost all measurements of the pulmonary flow were significantly different between days except PEP and PEP/ET. Mitral and tricuspid flow velocities (E_max_, E_mean_, A_max_, A_mean_, E_max_/A_max_) and E peak and A peak mitral and tricuspid VTI were significantly different between days.

Comparisons of the PW Doppler echocardiographic measurements of the aortic and mitral flows obtained from the right and from the left side are shown in Tables 1 and 2 respectively. For the aortic flow, only PEP and PEP/ET were not significantly different whilst all other parameters were significantly different from the right and the left side. Most of the parameters, especially V_max_ and V_mean_, were significantly higher when they were obtained from the left side than from the right side. For mitral flow, all parameters of A peak but only ET of E peak were not significantly different from both sides. The mitral E_max_, E_mean_ and VTI of E peak were higher when obtained from the left side than when obtained from the right side.

**Table 1:** Comparison of pulse wave Doppler echocardiographic parameters of the aortic flow obtained on the right and on the left side in 10 healthy adult Saanen goats, and within-day and between-day variability of these measurements performed on 3 following days by the same observer

	**LSMean ± SE Right side**	**LSMean ± SE Left side**	**Observed Mean ± SD**	**Confidence Intervals (5 – 95%)**	**Within-day CV (%)**	**Between-day CV (%)**
V_max_ (m/s)	0.99 ± 0.006	1.11 ± 0.006 *	1.05 ± 0.14	0.98 – 1.11	4.87	6.62
V_mean_ (m/s)	0.73 ± 0.004	0.79 ± 0.004 *	0.76 ± 0.09	0.72 – 0.81	5.60	6.43
ET (ms)	266.89 ± 1.41	271.95 ± 1.40 *	269.42 ± 23.17	254.38 – 284.45	4.68	6.37
VTI (cm)	19.58 ± 0.14	21.42 ± 0.14 *	20.51 ± 2.77	18.98 – 22.03	6.76	8.47
PEP (ms)	49.80 ± 0.55	50.89 ± 0.55	50.38 ± 6.84	44.48 – 56.28	11.49	13.35
TTP (ms)	102.53 ± 1.57	82.74 ± 1.56 *	92.58 ± 19.70	75.77 – 109.37	16.31	20.70
Acc slope (m/s^2^)	10.28 ± 0.24	13.95 ± 0.24 *	12.13± 3.29	9.58 – 14.67	17.67	23.93
Dec slope (m/s^2^)	6.35 ± 0.08	6.067 ± 0.08 *	6.21 ± 1.44	5.38 – 7.03	11.14	15.16
Dec Time (ms)	164.35 ± 1.79	189.21 ± 1.78 *	176.85 ± 26.82	157.68 – 196.01	10.30	12.36
SV (ml)	61.77 ± 0.45	67.61 ± 0.45 *	64.71 ± 8.60	59.87 – 69.55	6.76	8.52
CO (l/min)	5.80 ± 0.44	6.16 ± 0.44 *	5.98 ± 0.96	5.51 – 6.45	6.76	8.46
SI (ml/kg)	0.96 ± 0.007	1.05 ± 0.007 *	1.00 ± 0.14	0.92 – 1.08	6.76	8.98
CI (ml/kg/min)	89.81 ± 0.72	95.27 ± 0.72 *	92.45 ± 14.91	84.78 – 100.11	6.76	8.82
PEP/ET	0.19 ± 0.003	0.19 ± 0.003	0.19 ± 0.03	0.16 – 0.22	13.37	9.46

Acc: Acceleration, CI: Cardiac Index, CO: Cardiac output, CV: Coefficient of variation, Dec: Deceleration, ET: Ejection time of the aortic flow, LSMean: Least Square Mean, PEP: Pre-ejection period of the aortic flow, SD: Standard deviation, SE: Standard error, SI: Stroke index, SV: Stroke volume, TPP: Time to peak of the aortic flow, V_max_: Peak velocity of the aortic flow, V_mean_: Mean velocity of the aortic flow, VTI: Velocity time integral of the aortic flow.

* Significantly different from corresponding value obtained from the right side, p < 0.05.

**Table 2:** Comparison of pulse wave Doppler echocardiographic parameters of the mitral flow obtained on the right and on the left side in 10 healthy adult Saanen goats, and within-day and between-day variability of these measurements performed on 3 following days by the same observer

	**LSMean ± SE Right side**	**LSMean ± SE Left side**	**Observed Mean ± SD**	**Confidence Intervals (5 – 95%)**	**Within-day CV (%)**	**Between-day CV (%)**
*E-Peak*						
E_max_ (m/s)	0.57 ± 0.005	0.59 ± 0.005 *	0.58 ± 0.04	0.52 – 0.63	7.23	10.66
E_mean_ (m/s)	0.40 ± 0.004	0.42 ± 0.004 *	0.41 ± 0.03	0.37 – 0.45	7.97	10.80
VTI (cm)	8.84 ± 0.10	9.52 ± 0.10 *	9.18 ± 0.97	8.07 – 10.29	10.52	13.81
ET (ms)	224.57 ± 2.52	227.54 ± 2.52	226.06 ± 22.17	199.01 – 253.11	9.91	13.65
Dec Time (ms)	119.99 ± 1.95	104.99 ± 1.95 *	112.49 ± 19.12	91.56 – 133.41	17.75	21.22
Dec slope (m/s^2^)	5.17 ± 0.20	6.74 ± 0.20 *	5.96 ± 1.45	3.78 – 8.13	22.35	41.53
*A-Peak*						
A_max_ (m/s)	0.51 ± 0.005	0.52 ± 0.005	0.51 ± 0.05	0.46 – 0.56	10.24	11.50
A_mean_ (m/s)	0.39 ± 0.004	0.39 ± 0.004	0.39 ± 0.04	0.34 – 0.43	11.42	12.79
VTI (cm)	4.27 ± 0.06	4.26 ± 0.06	4.27 ± 0.64	3.61 – 4.92	14.98	17.43
ET (ms)	110.74 ± 1.44	110.44 ± 1.44	110.59 ± 14.76	95.17 – 126.01	13.26	15.90
E_max_/A_max_	1.12 ± 0.01	1.15 ± 0.01	1.14 ± 0.11	1.01 – 1.26	9.31	12.35

A_max_: peak velocity of the mitral flow during the atrial contraction, A_mean_: mean velocity of the mitral flow during the atrial contraction, CV: Coefficient of variation, Dec: Deceleration, E_max_: peak velocity of the mitral flow during the early ventricular filling, E_mean_: mean velocity of the mitral flow during the early ventricular filling, ET: Ejection time, LSMean: Least Square Mean, SD: Standard deviation, SE: Standard error, VTI: Velocity time integral.

* Significantly different from corresponding value obtained from the right side, p < 0.05.

The variability of all the PW Doppler measurements was evaluated on the basis of the within-day and between-day CV and of the calculation of confidence intervals. The results of the measurements of the aortic, mitral, pulmonary and tricuspid flows are shown in Tables 1, 2, 3 and 4, respectively. Most of the parameters had low to moderate variability excepted for TTP of the aortic flow, acc slope of the aortic and pulmonary flows, and Dec Time and Dec slope of the mitral and tricuspid E peaks that showed a high variability. For all flows, within-day variability was clearly lower than between-day variability.

**Table 3:** Within-day and between-day variability and confidence interval of pulse wave Doppler echocardiographic measurements of the pulmonary flow in 10 healthy adult Saanen goats performed 3 times at 1 day interval by the same observer

	**Observed Mean ± SD**	**Confidence Intervals (5 – 95%)**	**Within-day CV (%)**	**Between-day CV (%)**
V_max_ (m/s)	0.99 ± 0.12	0.93 – 1.05	5.33	6.19
V_mean_ (m/s)	0.76 ± 0.10	0.71 – 0.80	5.49	6.58
ET (ms)	266.7 ± 23.4	254.45 – 279.04	4.77	5.26
VTI (cm)	20.14 ± 2.74	19.02 – 21.25	6.03	6.31
PEP (ms)	51.1 ± 8.0	44.89 – 57.39	12.34	13.94
TTP (ms)	112.5 ± 27.1	95.57 – 129.44	15.89	17.17
Acc slope (m/s^2^)	9.31 ± 2.83	7.35 – 11.27	20.19	24.02
Dec slope (m/s^2^)	6.81 ± 1.97	5.71 – 7.91	12.54	18.36
Dec Time (ms)	154.3 ± 31.9	136.44 – 172.05	12.48	13.17
SV (ml)	77.69 ± 11.16	73.41 – 81.97	6.02	6.28
CO (l/min)	7.34 ± 1.50	6.93 – 7.74	6.02	6.30
SI (ml/kg)	1.20 ± 0.17	1.13 – 1.27	6.02	6.47
CI (ml/kg/min)	113.31 ± 22.88	106.82 – 119.80	6.02	6.53
PEP/ET	0.19 ± 0.04	0.16 – 0.22	14.86	16.41

Acc: Acceleration, CI: Cardiac index, CO: Cardiac output, CV: Coefficient of variation, Dec: Deceleration, ET: Ejection time of the pulmonary flow, PEP: Pre-ejection period of the pulmonary flow, SD: Standard deviation, SI: Stroke index, SV: Stroke volume, TPP: Time to peak of the pulmonary flow, V_max_: Peak velocity of the pulmonary flow, V_mean_: Mean velocity of the pulmonary flow, VTI: Velocity time integral of the pulmonary flow.

**Table 4:** Within-day and between-day variability and confidence interval of pulse wave Doppler echocardiographic measurements of the tricuspid flow in 10 healthy adult Saanen goats performed 3 times at 1 day interval by the same observer

	**Observed Mean ± SD**	**Confidence Intervals (5 – 95%)**	**Within-day CV (%)**	**Between-day CV (%)**
*E-Peak*				
E_max_ (m/s)	0.67 ± 0.15	0.60 – 0.73	9.82	10.34
E_mean_ (m/s)	0.49 ± 0.12	0.44 – 0.54	10.11	10.88
VTI (cm)	10.60 ± 3.10	9.14 – 12.06	15.21	15.69
ET (ms)	220.6 ± 54.5	196.34 – 244.77	11.16	12.53
Dec Time (ms)	108.9 ± 34.7	86.23 – 131.72	22.03	23.81
Dec slope (m/s^2^)	6.89 ± 3.09	5.02 – 8.74	25.65	30.87
*A-Peak*				
A_max_ (m/s)	0.58 ± 0.12	0.51 – 0.64	10.70	12.29
A_mean_ (m/s)	0.45 ± 0.10	0.39 – 0.49	11.07	12.60
VTI (cm)	5.11 ± 1.24	4.30 – 5.91	16.20	17.97
ET (ms)	117.3 ± 24.2	103.86 – 130.77	11.41	13.08
E/A	1.16 ± 0.19	1.03 – 1.29	11.14	12.35

A_max_: peak velocity of the tricuspid flow during the atrial contraction, A_mean_: mean velocity of the tricuspid flow during the atrial contraction, CI: Confidence Intervals, CV: Coefficient of variation, Dec: Deceleration, E_max_: peak velocity of the tricuspid flow during the early ventricular filling, E_mean_: mean velocity of the tricuspid flow during the early ventricular filling, ET: Ejection time, SD: Standard deviation, VTI: Velocity time integral.

## Discussion

The results of this paper show that good quality PW Doppler flow spectrum of each cardiac valve can be obtained on standing unsedated goats like in most other animal species. The effects of the physiological factors such as body size [20], breed [21], age [22], lactation [18] and pregnancy [18,23], which are known to affect PW Doppler measurements were not investigated in the present study. A colour-flow Doppler echocardiography performed each day before each PW-Doppler echocardioghraphic protocol did not reveal any abnormality. Particularly, any suspicion of interatrial communication or patent foramen ovale such as reported in 9 healthy Saanen goats [17] could be excluded.

To assess a pulmonary spectrum flow in this study, the transducer had to be advanced far cranially under the forelimb, which required an assistant pulling the right forelimb forward and upward during the examination. This procedure was not well tolerated by all of the investigated animals, and implies than the investigated goat is fully accustomed to being handled, which is not always the case in caprine field practice. Moreover, at this level, the cardiac window often appeared to be narrow as previously reported in goats [16,18]. But when a heart base view of sufficient quality was obtained, the recorded pulmonary outflow velocity spectrum was generally speaking of good quality, suggesting a good alignment of the transducer with the pulmonary blood flow, as previously reported in horses [10,24]. On the contrary, the tilted 2D right parasternal long axis four chambers view was in most of the goats of very good quality, but the obtained flows at the level of the mitral and tricuspid valves were generally underwhelming, as described in horses [7]. Moreover, a true left parasternal apical view as used in dogs to measure aortic, mitral and sometimes tricuspid flows [10,25], was not possible to obtain in goats because of the presence of gas in the reticulo-rumen [18].

The repeatability of the PW Doppler measurements in our study was poor, especially for Acc and Dec slopes of all intra-cardiac blood flows and, on the contrary to what could be expected on the basis of the good quality of the pulmonary flow spectrum, for all pulmonary flow measurements. PW Doppler measurements have been already described as poorly repeatable in other species [10,26,27]. Moreover, slopes of intra-cardiac blood flows are generally the less repeatable Doppler parameters [28]. Aortic and pulmonary flow measurements were previously often reported as repeatable measurements [25,28,29], except in one study on horses in which they were poorly repeatable [27]. The lack of repeatability of the pulmonary flow in the studied goats could be explained by the difficulty to obtain a good quality 2D image of the right ventricular outflow tract, then to place the sample volume day to day exactly at the good location. Recordings of the pulmonary flow from a left parasternal cranial long axis right ventricular outflow view or from a left parasternal short axis view with aorta and pulmonary artery, as used in small domestic animals [10,25,29], has not been investigated in this study but appeared to be difficult to obtain in goats.

The variability of the PW Doppler measurements in our study was quite high, but was in accordance with previous studies in dogs, cats and horses [7,25,26,29]. Independently of the valve where the blood flow was measured, the within-day and between-day CV ranged from 4.68% to 41.53%, but most of these were inferior to 20% (Tables 1, 2, 3, 4). The main source of variability could be the poor alignment of the transducer with the direction of the blood flow [10,26,27]. Measurements of the Acc or Dec slopes and times from the different blood flows were the most variable parameters. This is in agreement with previous studies in horses [26] and in dogs [25].

In the studied goats, the mean values of the mitral velocity spectrum obtained from a tilted left parasternal long axis four chamber view were significantly different from those obtained from a tilted right parasternal long axis four chamber view, except for all parameters of the A peak and for ET of the E peak. Moreover, E_max_ and E_mean_ were lower when the measurements were performed from the right side than from the left side, which suggests that in goats, the mitral flow should be interrogated from the left rather than from the right hemi thorax. This result is in agreement with previous studies on other domestic animals since, to record the mitral flow, a tilted left parasternal long axis four chambers view is recommended in horses [7], and a left parasternal apical view is recommended in sheep [9] and in dogs [4].

The E_max_/A_max_ ratio is a parameter often used to evaluate the left ventricular diastolic function in man [2,30]. Independently of the side from which it is measured, the E_max_/A_max_ ratio of the mitral flow was rather similar to the tricuspid flow E_max_/A_max_. On the contrary to what was reported in sheep [9], in most goats of this study, E_max_was higher than A_max_ for both mitral and tricuspid flows, and only one goat had E_max_/A_max_ < 1 for mitral flow obtained from the right side. The same was observed in 8 of 40 investigated healthy horses [7] and was explained as a more accurate alignment of the transducer with the A wave of atrial contraction than with the E wave of the early rapid ventricular filling. Measurements of E peak and A peak seemed also to depend on HR. In goats as in sheep, it has been reported than the A peak is closer to the E peak with increasing HR, and when HR was more than 120 beats/min, fusion of the two peak can occur [9].

Measurements of aortic velocity spectrum are very interesting because they allow assessing left ventricular SV and CO [2]. In this study, except for PEP and PEP/ET, the aortic velocity spectrum measurements obtained from the tilted left parasternal long axis five chambers view were significantly higher than those obtained from a tilted right parasternal long axis five chambers view. This is in agreement with the results obtained in horses [7], and could be explained by a better alignment between the transducer and the blood flow from the left hemi thorax. This could also explain why previous measurements of aortic flow parameters reported in Swedish goats and obtained from the right side [18] were lower than those obtained in this present study.

## Conclusions

PW-Doppler flow parameters reference values in adult, healthy, non-pregnant, female goats of the Saanen breed are reported in this study. As documented in other species, goats’ PW Doppler parameters measurements showed a poor between-day repeatability and a high but acceptable within-day and between-day variability. Tricuspid and pulmonary flows should be evaluated from the right hemi thorax whereas mitral and aortic flows should be obtained from the left hemi thorax. Moreover, in the future, it could be interesting to evaluate the effect of physiological factors in order to establish fully documented PW Doppler echocardiographic reference values in goats.

## Competing interests

The authors declare that they have no competing interests.

## Authors´ contributions

Conception and design of the study: AAL, FF and HA; Data acquisition: AAL; statistical analysis: AAL, FF; Drafting and critically revising the manuscript: AAL, FF, MLM, CFS, SD and HA. All authors read and approved the final manuscript.
